# Development of humanistic nursing practice guidelines for stroke patients

**DOI:** 10.3389/fpubh.2022.915472

**Published:** 2022-08-09

**Authors:** Min Li, Yu-gui Ji, Zi-qing Yang, Hong-zhen Xie

**Affiliations:** ^1^Department of Trauma Surgery, Chongqing Emergency Medical Center, Chongqing University Central Hospital, Chongqing, China; ^2^Department of Neurosurgery, General Hospital of Southern Theatre Command, Guangzhou, China; ^3^Department of Health Medicine, General Hospital of Southern Theatre Command, Guangzhou, China

**Keywords:** stroke, humanistic care, humanistic nursing, practice guidelines, Delphi technique

## Abstract

**Purpose:**

To construct humanistic nursing practice guidelines suitable for stroke patients.

**Design:**

This study was a development and validation study of guidelines using multiple methods, including literature review, qualitative research, questionnaire survey, thematic discussion, and Delphi expert consultation.

**Methods:**

Twenty-five experts from seven provinces and municipalities in China were interviewed over two rounds from December 2020 to February 2021. The first-level index was scored for importance and rationality, while the second-level index was scored for importance and feasibility using a five-point Likert scale. Delphi data was collected *via* a paper version of the questionnaire. The coefficients of variation and coordination were used to represent the degree of dispersion of expert opinions.

**Findings:**

In the two rounds of letter consultation, the questionnaire's recovery and effective rates were both 100%, while the opinion submission rates of the two rounds were 84 and 52%, respectively. Moreover, the expert authority coefficient was 0.91, and the coordination coefficients of expert opinions in the first round were as follows: importance of 0.03 and rationality of 0.07 for the first-level index; importance of 0.09 and feasibility of 0.11 for the secondary index. In round two, the coordination coefficients of expert opinions were as follows: importance of 0.04 and rationality of 0.05 for the first-level indicators; importance of 0.12 and feasibility of 0.10 for the secondary index. The results for the secondary index were *P* < 0.001 for the two rounds. The humanistic nursing practice guidelines for stroke patients that were ultimately formed included five first-level indicators (physiological care, safety care, emotional care, dignity care, and rehabilitation needs) and 46 s-level indicators.

**Conclusion:**

Our results show that the “Practice Guidelines for Humanistic Nursing for Stroke” established by experts adopts Maslow's hierarchy of needs as its structural framework. It meets people's basic needs and can provide a reference for the construction of a humanistic nursing specialty practice for stroke patients.

**Clinical relevance:**

Humanistic nursing guidelines for stroke could provide a reference for the construction of humanistic nursing practice in the stroke specialty.

**Clinical resources:**

Copeptin and long-term risk of recurrent vascular events after transient ischemic attack and ischemic stroke: population-based study https://pubmed.ncbi.nlm.nih.gov/26451023/. Effectiveness and usage of a decision support system to improve stroke prevention in general practice: a cluster randomized controlled trial https://pubmed.ncbi.nlm.nih.gov/28245247/. Guidelines for adult stroke rehabilitation and recovery: a guideline for healthcare professionals from the American Heart Association/American Stroke Association https://pubmed.ncbi.nlm.nih.gov/27145936/.

## Introduction

Stroke is one of the three leading causes of disease-related death in humans (1, 2) and the leading cause of death in China (3). The incidence, disability, recurrence, and death rates of this disease remain high. The number of stroke survivors at any one time is around 11 million, and there are ~2.4 million first-time stroke patients in China each year. Stroke also accounts for roughly 1.1 million fatalities annually in China (1, 3). The neurological impairments and changes in self-roles experienced by stroke patients lead to a sudden increase in psychological pressure (4). These neurological deficiencies and self-role alterations, which can include shame, anxiety, despair, and other unpleasant feelings, were found to become more evident as the psychological stress of stroke patients rose (5). Additionally, a spectrum of mental disorders and severe psychiatric conditions may develop after a stroke that can lead to self-harm, suicide, or other social issues, significantly affecting patients' quality of life (6, 7) and imposing a heavy burden both on their family and society (4, 8).

In the West, the term “human nature” is derived from the Latin “man,” indicating that the concept is focused on people. In China, however, the analogous term comes from the Book of Changes, which views mending and saving as the most advantageous concept (9). Medical personnel naturally transfer this idea of caring for the “whole person” to patients in vulnerable states, transforming the professional connection into one that is more family-like and demonstrating behavior that ensures patients feel loved and cared for (10). The Chinese approach places a strong emphasis on respect for human life and the development of humanistic qualities among medical staff, built on the foundation of complete professional knowledge. This spirit should be incorporated into clinical practice to ensure that patients are treated promptly and effectively, that their discomfort is removed or lessened, and that they are treated with appropriate respect and compassion.

Nursing humanistic care guidelines were developed in 2003 with the support of the International Association for Circular Nursing (11). The ANCM model was proposed in the same year with the goal of increasing nursing capacity by offering programs to nurses that include nursing evaluation, planning, and the maintenance of continuity of care (12). Studies have shown that effective psychological intervention and humanistic care can improve stroke patients' mental health status, significantly reduce the incidence of mental disorders after stroke, avoid the range of adverse events that can be caused by psychological problems, and directly affect rehabilitation outcomes (13). Humanistic treatment may influence rehabilitation outcomes among stroke patients, as well as their physical and emotional wellbeing (14). It is accordingly a crucial component of raising the quality of the nursing care provided in the stroke unit.

However, no unified evaluation standard for humanistic medical care or practical guidance for specialized care is provided in China. Nursing staff provide care based largely on their own awareness and cultivation (3, 4). As a result, patients' immediate care needs cannot be fully met, suggesting that the quality of practice needs to be further improved (3, 15). In foreign countries, the theory of humanistic care has been studied in depth and is widely applied. The practice of humanistic nursing focuses primarily on studying the rules for the implementation of operable humanistic care, with nurse care practice as the focus, and concentrates more on evaluation of the care effect than on the structure and process of the care. Notably, there is still a lack of nursing practice standards in place governing care for stroke patients (16). Therefore, this research highlights the difficulty and pain points of humanistic nursing practice from the domestic medical background and stroke wards, based on theoretical analysis and using the practical demands of cerebral apoplexy patients and their families as a guide. Rigorous research methods were employed to construct comprehensive humanistic nursing college practice guidelines for cerebral apoplexy patients and provide a model reference for holistic care.

## Materials and methods

### Study design

**Figure 1:**
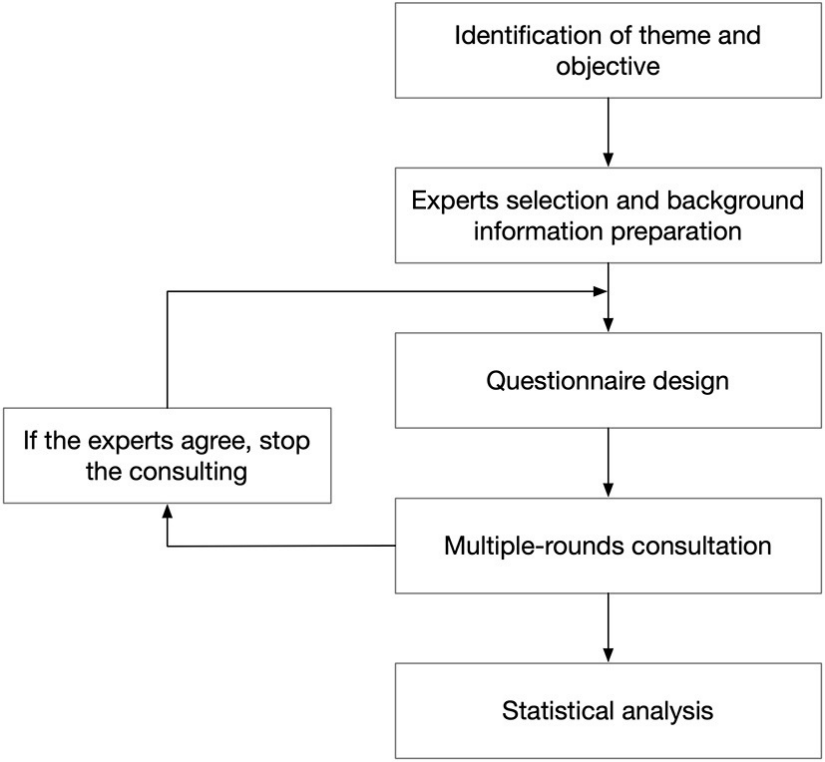
The flow diagram of the study.

This research aims to develop humanistic nursing practice recommendations for stroke patient care that are both practical and scientific. The humanistic nursing practice questions developed for stroke patients were devised following a literature review, qualitative study, questionnaire survey, and theme discussion, then added to the program item pool. The “Practice Guidelines for Humanistic Nursing for Stroke” program, which contains five dimensions and 45 elements, was created as a preliminary result of the study team's evaluation of nurses, stroke patients, and results (17). A research team was formed to continually enhance and perfect the software based on the original case practice. The humanistic nursing practice guiding program for stroke was developed using the Delphi expert consultation approach. The processes utilized in the present study are presented in Figure 1.

The research team consisted of 15 people, including the director of the nursing department, one deputy director, the director of the nursing department in charge of scientific research, and six nursing master's graduate students who had been working in the clinical setting as specialist stroke practitioners for an extended period (such as clinical nurses, experts in nursing and medical management, technicians skilled in rehabilitation, stroke specialist nurses, etc.). The group comprised people in five senior, five intermediate, and five primary positions. The key responsibilities of the team members were literature search, expert selection, expert letter questionnaire creation, dissemination, recovery, opinion analysis, data summarization, and program enhancement. Internal group meetings were conducted to assess the appropriateness of the questionnaire before it was distributed to experts.

### Components of letter questionnaire

In brief, the questionnaire comprised a setting preface, general information, and the scheme content evaluation table. In the setting preface, the background, purpose, and significance of the research are elaborated in detail, and information about filling in the questionnaire and the time cut-off point is also provided. Based on the suggestions of patients and their families, the experts' questionnaire was constructed, containing general information about the experts' age, educational background, professional title, years worked, field of work, research direction, familiarity with the topic, and the basis of judgment for the self-evaluation. The scheme content evaluation table was set up as follows: the first-level index was scored for importance and rationality, while the second-level index was scored for importance and feasibility. The scoring standard was a five-point Likert scale, and points were scored from five to one in order according to relevance: very important (five points), important, neutral, not very important, and not at all important (one point). Rationality and feasibility scores refer to the score's importance.

### Expert selection and survey

We used the judgment sampling method in the present study. Criteria were established for the selection of experts who met the inclusion conditions. The responses of eligible participants who consented to participate were included in this survey. The qualifications for experts were as follows: 10 years or more experience in nursing, medical treatment, and management of stroke wards, or 10 years or more in the fields of humanities nursing, education, and research; those who continue to pay attention to stroke or humanistic care and are familiar with the research progress and current status in this field; or those with a senior technical title and a clinical front-line expert title at an intermediate level or above (intermediate title: ≤ 10% of the total number of experts). Participants were voluntary and active.

At this stage, the grading of the “Practice Guidelines for Humanistic Nursing for Stroke” scheme and the collection of suggestions for modification were achieved by sending out questionnaires to experts in the form of letters. The distribution and collection of the consultation questionnaires were carried out by combining online and offline methods. The research team sorted out opinions received, generated statistical data, modified the scheme, etc., and then conducted the next round of letter consultation based on the revised draft. The letter consultation ceased when the opinions obtained from experts were unanimous. If errors or gaps were found in the expert questionnaire, we sent emails to experts to verify whether the content represented the real opinions of the experts or clerical errors. If more than 10% of the items in the questionnaire were not evaluated, the expert would be disqualified for letter consultation in the next round and the questionnaire would be removed from the sample; fortunately, there was no miswriting in this study, and the validity and integrity of the questionnaires collected in the two rounds of correspondence consultation were found to be 100%.

### Data analysis

SPSS 21.0 was used for data statistics and analysis. Frequency and percentage are used to describe the basic information, such as academic background, professional title, and the experts' research direction. The number and speed of the experts' responses to the questions, as well as their excitement, are expressed as a ratio. The concentration of expert viewpoints is shown in terms of the mean value, full mark rate, and selection rate. The degree of expert opinion dispersion is represented by the covariance and coordination coefficients. Expert authority coefficient (CR) is calculated from the average of the experts' judgment basis (Ca) and familiarity degree (CS) of the research scheme, i.e., CR = (Ca + CS) / 2.

## Results

### Basic information of experts

A total of 25 experts were included in this study, hailing from various different medical institutions and scientific research institutions both in and outside of Guangdong, including six in Guangdong Province and 19 outside Guangdong Province (Shanghai, Chongqing, Zhengzhou, Wuhan, Suzhou, and Jinan). The experts worked in medical, nursing, rehabilitation, and other multidisciplinary fields, including cerebrovascular disease, stroke rehabilitation, stroke psychology, humanistic care, nursing management, and nursing education, among others. The median expert age was 49.72 ± 9.04 years old, while the median number of years working in their research field was 29.04 ± 8.84 years (Table 1).

**Table 1:** General information of experts (*n* = 25).

**Items**	**Number**	**Composition ratio (%)**
Gender		
Male	2	8
Female	23	92
Degree		
Bachelor degree	9	36
Master degree	10	40
Doctor degree	6	24
Rank		
Middle	2	8
Senior	23	92
Working fixed number of year		
10~	6	24
20~	5	20
30~	12	48
40~	2	8
Have relevant work experience in stroke department		
Yes	23	92
No	2	8
Have experience in humanities studies, humanities education or training		
Yes	25	100
No	0	0
Fields of expertise		
Medical or nursing management	17	68
Clinical first-line (nursing)	12	48
College teachers	3	12
First-line clinical (medical)	1	8
Clinical first-line (technology)	1	4
Research direction of experts		
Cerebrovascular disease	15	60
Stroke rehabilitation	12	48
Humanity care	12	48
Nursing management	11	44
Nursing education	6	24
Stroke psychology	2	8

### Positive coefficient and degree of opinion authority

Questionnaire return and expert response are both positive coefficients. In this study, a total of two rounds of letter consultations were conducted, and the effective recovery rate of questionnaires was 100% (Table 2). Thirty-four people (21 in the first round and 13 in the second) gave written suggestions in the two rounds. The rate of opinions given was 84 and 52%, respectively, indicating highly active participation on part of the experts. The expert authority coefficient (CR) was calculated from the mean of the experts' judgment basis (CA) and familiarity degree (CS) of the research scheme, i.e., CR = (0.93 + 0.89) / 2 = 0.91. It is generally considered that CR ≥ 0.7 is highly credible, and that the higher the score, the higher the degree of credibility; thus, it can be inferred that the data reliability of the letter consultation results is on the higher end.

**Table 2:** Questionnaire collection and expert response.

**Consultation round**	**Total distribution**	**Total recovery**	**Recovery (%)**	**Effective rate (%)**	**Response rate (%)**	**Proposal rate (%)**
Round 1	25	25	100	100	100	84
Round 2	25	25	100	100	100	52

### Degree of concentration and coordination of opinions

Experts evaluated the dimensions of the “Humanistic Nursing Practice Guidelines for Stroke” in terms of their importance and rationality, and the items were evaluated based on their feasibility and importance. The scoring standard was a five-point Likert scale, with five points assigned to the highest score and one point to the lowest. The importance and rationality of each dimension's calculated scores are above 4.80, while the importance and feasibility of the practice item scores are above 4.20; moreover, the variation coefficient, in addition to the entry D8, are <0.25, completing the entire process of inquiry. It can be concluded that expert opinion is relatively uniform, it is better to write to inquire by letter. See Table 3 for details.

**Table 3:** Kendall coordination coefficient.

**Items**	**Round 1(*****n*** = **25)**				**Round 2(*****n*** = **25)**			
	**First-level indicator**		**Secondary indicators**		**First-level indicator**		**Secondary indicators**	
	**Importance**	**Rationality**	**Importance**	**Feasibility**	**Importance**	**Rationality**	**Importance**	**Feasibility**
W value	0.03	0.07	0.12	0.10	0.04	0.05	0.09	0.11
X2 value	2.86	6.67	128.15	105.93	4.29	5.20	100.33	117.66
*P*-value	0.58	0.16	<0.001	<0.001	0.37	0.27	<0.001	<0.001

### Data summary and analysis

#### Round 1

This round of letter consultation received 95 written suggestions from 21 experts, involving 36 items, of which 29 items were written suggestions while seven were structural suggestions. According to the experts' opinions, following comprehensive discussion by the research group, one first-level indicator and 25 language descriptions were modified. Three indicators were adjusted in dimension, three secondary indicators were combined, and one was split. There are five new caring targets of the first-level index and eight new caring targets of the second-level index. A total of three second-level indicators were also deleted.

The first-level indicators can be described as follows. (1) The importance score was between 4.88 and 5.00, with a mean score of 4.95; the full score rate was 92–100%, with a mean of 96%. The coefficient of variation ranged from 0 to 0.09, with a mean of 0.04. The item with the highest score (100%) was life care, while the item with the lowest score (92%) was rehabilitation care. (2) The rationality scores ranged from 4.80–4.96, with a mean score of 4.91; the full score rate was 84–96%, with a mean of 92%. The coefficient of variation ranged from 0.04 to 0.06, with a mean of 0.06. The items with the highest score (96%) were emotional and dignity care, while the item with the lowest score (84%) was rehabilitation care (Appendix 1).

The second-level indicators can be described as follows. (1) The importance score was between 4.28 and 5.00, with a mean score of 4.83; the full score rate was 60–100%, with a mean of 85.33%. The coefficient of variation ranged from 0 to 0.27, with a mean of 0.08. The only item ≥0.25 was D8, and the coefficient of variation ranged from 0 to 0.20 after the deletion of this item. The items with the highest score (100%) were A3, B1, B2, and B6, while the items with the lowest score (60%) were C8 and D8. (2) Feasibility scores ranged from 4.20 to 4.92, with a mean score of 4.64, along with a rate of 40–92% and a mean rate of 70.22%. The coefficient of variation ranged from 0.06 to 0.23, with a mean of 0.12. The item with the highest score (92%) was D5, while the item with the lowest score (40%) was E9. Details can be found in the online supplement (Appendix 2).

#### Round 2

In this round of letter consultation, 13 experts provided text suggestions. There were a total of 42 recommendations for improvement; 41 of these were suggestions for expression and modification, while one pertained to structure. The expert opinions were found to be relatively centralized and consistent. In full reference to the expert opinions, all research group members were organized for many discussions. After repeated deliberation, the final draft of “Humanistic Nursing Practice Guidelines for Stroke” was established, including five dimensions and a total of 46 items.

The first-level indicators can be described as follows. (1) The importance score ranged from 4.84 to 4.96, with a mean score of 4.92; the full score rate was 84–96%, with a mean of 92%. The coefficient of variation ranged from 0.04 to 0.08, with a mean of 0.05. The items with the highest score (96%) were life and dignity care, while the item with the lowest score (84%) was emotional care. (2) The rationality scores ranged from 4.84 to 4.96, with a mean score of 4.90; the full score rate was 84–96%, with a mean score of 90%. The coefficient of variation ranged from 0.04 to 0.08, with a mean of 0.06. The item with the highest score (96%) was dignity care, while the item with the lowest score (88%) was safety care (Appendix 1).

The second-level indicators can be described as follows. (1) Importance score was between 4.68 and 5.00, with a mean of 4.90; the full mark rate was 72–100%, with a mean of 90.35%. The coefficient of variation ranged from 0 to 0.13, with a mean of 0.06. The items with the highest score (100%) were A2, B4, B8, B9, D1, and D2, while the item with the lowest score (72%) was C8. (2) Feasibility score ranged from 4.40 to 5.00, with a mean of 4.78; the full score rate was 52–100%, with a mean of 79.48%. The coefficient of variation ranged from 0 to 0.17, with a mean of 0.09. The item with the highest score (100%) was D2, while the item with the lowest score (52%) was C8. Details can be found in the online supplement (Appendix 2).

## Discussion

Humanistic care respects the patient's life values, personality, and personal privacy at its core. Humanistic care for patients is a creative, personalized, holistic, and practical nursing tool. It acts as a bridge between nurses and patients, improving the quality of nursing available to patients who have suffered from stroke (18). This study aimed to establish a humanistic nursing module for stroke patients, using literature retrieval and Delphi expert consultation.

### Reliability of the results of correspondence inquiries

The “Humanistic Nursing Practice Guidelines for Stroke” was established based on two rounds of expert letter consultation. Across the entire consultation process, the expert authority coefficient (CR) was 0.91, and the questionnaire's effective recovery rate was 100%.

A total of 34 people were recruited (21 in the first round and 13 in the second). Improvement suggestions from the two rounds numbered 95 and 42, or 84 and 52%, respectively. The W values of importance and practicability for the practice items were 0.12, 0.10, 0.09, and 0.11, respectively.

The above data show that experts display a good level of enthusiasm, the expert opinions tend to be consistent, the letter consultation results are reliable, and the results of the letter consultation are effective overall. The selected experts are representative of their regions, professional fields, and research directions. The 25 experts were from the east, west, south, north, and central regions of China and worked at a number of medical and scientific research institutions, including six in Guangdong Province and nine from outside of Guangdong Province (Shanghai, Chongqing, Zhengzhou, Wuhan, Suzhou, and Jinan). Experts were involved in numerous professional fields, including medical, nursing, rehabilitation, and other multidisciplinary practices, and had expertise in areas including cerebrovascular disease, stroke rehabilitation, stroke psychology, humanistic care, nursing management, and nursing education, among others. The mean number of years worked in their field is 29.04 ± 8.84 years. Eleven experts (44%) are very familiar with the research field, while 14 experts (56%) describe themselves as knowledgeable about this research field. It can be determined that the experts involved in the letter consultation have rich experience and high familiarity with the research topic, indicating that the results of the letter consultation are reliable.

### The scientific nature of the humanistic nursing practice guidance scheme for stroke

#### From a formation process perspective

This study conducted qualitative interviews with doctors, nurses, and rehabilitation technicians in stroke wards. Here, the goal was to understand the current situation of humanistic nursing in those wards from different perspectives, as well as to identify the entry point for the construction of humanistic nursing practice guidelines, based on the literature review and the previous research conducted by the research group. By learning from stroke patients and their families, the humanistic nursing measures could be explored based on the actual care needs and real-life experiences of these patients. Moreover, the investigations of nurses working in stroke specialty were used to understand the clinical nurses' perspectives regarding the specific work of their own humanistic nursing perception and practical care practices, which further enriched the content of the program. After the symposium, the first draft of “Humanistic Nursing Practice Guidelines for Stroke” was discussed and revised, and the program was initially built. Following evaluations by the stroke ward nurses and their patients, the importance of the items included in the program was further revised, and a stroke humanistic nursing practice program was devised, using Maslow's hierarchy of needs as the structural framework. Through the application of the Delphi expert consultation method, two rounds of expert letter consultations regarding the draft plan were conducted to establish the “Humanistic Nursing Practice Plan for Stroke.” The process of program formation is closely linked; practical items were drawn from the guiding literature, along with the actual feelings and needs of the program practitioners and audience. The overall design is thus more aligned with the reality of the situation.

#### From a structural framework and content design perspective

The humanistic nursing practice guidance scheme for stroke developed in this study adopts Maslow's hierarchy of needs, a classic theory of behavioral psychology, as its structural framework, as this approach is in line with people's basic needs as well as universal in its structural design. The program structure can be summarized with reference to the following components. (1) *Physiological care*: maintain basic physiological needs, maximize patients' comfort, relieve pain, and preserve life; (2) *Safety care*: create a safety management culture for stroke patients to maximize the sense of security among patients and their relatives; (3) *Emotional care*: build an emotional support system that integrates the patient, family, social and medical interactions, using nurses as a link, so that patients can maximize their feelings of love and attention; (4) *Dignity care*: practice the concept of human-based nursing, so that patients feel the value of life and their individual and personal dignity are respected and treated equally; (5) *Rehabilitation care*: help patients improve their ability to return to family and society and maximize the improvement of the patients' life and their overall quality of life. In terms of content, the program combines domestic and foreign guidelines on stroke, expert consensus, evidence summaries, and nursing routines, among other materials (19–27), and refers to the opinions and suggestions offered by the users and recipients of this program, making it more scientific in terms of its content design.

#### Clinical implications

The secret to increasing nursing standards in stroke wards is people-oriented management. The doctor–patient ratio in China is currently unbalanced, rehabilitation is imperfect, and nursing time for patients is constrained. Thus, it is important to optimize the medical staff and create appropriate specialist positions in line with the unique attributes of the stroke specialty (e.g., trained nurse, rehabilitation trainer, psychotherapist, etc.). The organizational culture should be shaped alongside the establishment of a shared definition of motivation. These dedicated guidelines will provide reference for the construction of humanized nursing and will be beneficial to improving the effect of nursing for stroke patients in clinical practice.

#### Limitations

The present study has several limitations. First, this is a phenomenological study based on questionnaires and sampling. Despite careful quality control to minimize potential bias, our results may be vulnerable to the researchers' subjectivity. Second, our relatively small sample size may give rise to selection bias. Hence, further large and more strictly designed studies are warranted to validate our results.

## Conclusion

In conclusion, humanistic nursing guidelines for stroke were established and evaluated through two rounds of expert consultation. In future work, attention should be paid to the integration of humanistic management and practical needs, which could provide a reference for the construction of humanistic nursing practice for stroke patients.

## Data availability statement

The original contributions presented in the study are included in the article/Supplementary material, further inquiries can be directed to the corresponding author.

## Ethics statement

The studies involving human participants were reviewed and approved by General Hospital of Southern Theater Command. The patients/participants provided their written informed consent to participate in this study.

## Author contributions

ML and H-zX: conception and design. Y-gJ: administrative support. Z-qY: provision of study materials or patients. ML: collection and assembly of data, data analysis and interpretation, and manuscript writing. All authors approved the final manuscript.

## Funding

This study was conducted by the Health Care Project of the Whole Army (Project No. 16BJZ58), Science and Technology Project of Guangzhou Municipality (Project No. 201704020155), and supported by: (Project No. [2018]17 of Guangdong Education and Research Institute).

## Conflict of interest

The authors declare that the research was conducted in the absence of any commercial or financial relationships that could be construed as a potential conflict of interest.

## Publisher's note

All claims expressed in this article are solely those of the authors and do not necessarily represent those of their affiliated organizations, or those of the publisher, the editors and the reviewers. Any product that may be evaluated in this article, or claim that may be made by its manufacturer, is not guaranteed or endorsed by the publisher.
