# Supplementary material for: Development of humanistic nursing practice guidelines for stroke patients
Source: Front Public Health. 2022 Aug 9;10:915472. doi: 10.3389/fpubh.2022.915472 (PMC9395746; doi:10.3389/fpubh.2022.915472)
Supplement: Supplementary file 1 [file Data_Sheet_1.docx]

| **Appendix 1 Data statistics of the letter consultation (first-level indicator)** | | | | | | | | |
| --- | --- | --- | --- | --- | --- | --- | --- | --- |
| Index content | Importance | | | | [Rationality](D:/D%E5%AE%89%E8%A3%85%E5%8C%85/Dict/8.9.6.0/resultui/html/index.html#/javascript:;) | | | |
|  |  | S | CV | Full mark rate（%） |  | S | CV | Full mark rate（%） |
| First round | | | | | | | | |
| Life care | 5.00 | 0.00 | 0.00 | 100 | 4.92 | 0.28 | 0.06 | 92 |
| Safety care | 4.96 | 0.20 | 0.04 | 96 | 4.92 | 0.28 | 0.06 | 92 |
| Emotional care | 4.96 | 0.20 | 0.04 | 96 | 4.96 | 0.20 | 0.04 | 96 |
| Dignity care | 4.96 | 0.20 | 0.04 | 96 | 4.96 | 0.20 | 0.04 | 96 |
| Rehabilitation care | 4.88 | 0.44 | 0.09 | 92 | 4.80 | 0.50 | 0.10 | 84 |
| Second round |  |  |  |  |  |  |  |  |
| Life care | 4.96 | 0.20 | 0.04 | 96 | 4.92 | 0.28 | 0.06 | 92 |
| Safety care | 4.92 | 0.28 | 0.06 | 92 | 4.88 | 0.33 | 0.07 | 88 |
| Emotional care | 4.84 | 0.37 | 0.08 | 84 | 4.84 | 0.37 | 0.08 | 84 |
| Dignity care | 4.96 | 0.20 | 0.04 | 96 | 4.96 | 0.20 | 0.04 | 96 |
| Rehabilitation care | 4.92 | 0.28 | 0.06 | 92 | 4.92 | 0.28 | 0.06 | 92 |
