# Supplementary material for: Development of humanistic nursing practice guidelines for stroke patients
Source: Front Public Health. 2022 Aug 9;10:915472. doi: 10.3389/fpubh.2022.915472 (PMC9395746; doi:10.3389/fpubh.2022.915472)
Supplement: Supplementary file 2 [file Data_Sheet_2.docx]

| **Appendix 2** **Data statistics of the letter consultation (second-level indicator)** | | | | | | | | | | |
| --- | --- | --- | --- | --- | --- | --- | --- | --- | --- | --- |
| NO. | Items | Importance | | | | | [Feasibility](D:/D%E5%AE%89%E8%A3%85%E5%8C%85/Dict/8.9.6.0/resultui/html/index.html#/javascript:;) | | | |
|  |  |  | S | CV | Full mark rate（%） |  | | S | CV | Full mark rate（%） |
| First round | | | | | | | | | | |
| A1 | Strengthen predictive care, at all times will save the lives of patients in the first place | 4.84 | 0.37 | 0.08 | 84 | 4.60 | | 0.58 | 0.13 | 64 |
| A2 | Accurate identification of stroke symptoms, real-time observation of changes in the condition, pay attention to the patient's feedback on the condition | 4.92 | 0.28 | 0.06 | 92 | 4.76 | | 0.60 | 0.13 | 84 |
| A3 | The stroke green channel is activated with one key, and the medical team is harmonious and cooperative, and the whole process of treatment is smooth | 5.00 | 0.00 | 0.00 | 100 | 4.76 | | 0.44 | 0.09 | 76 |
| A4 | The feeding and nutritional status of patients were assessed dynamically, and targeted dietary guidance and oral feeding strategies were developed based on the evaluation results | 4.96 | 0.20 | 0.04 | 96 | 4.72 | | 0.61 | 0.13 | 80 |
| A5 | Provide bed defecation skills, diapers and other auxiliary equipment, nursing equipment improvement according to the needs of patients when necessary | 4.88 | 0.33 | 0.07 | 88 | 4.92 | | 0.28 | 0.06 | 92 |
| A6 | Minimize unnecessary catheter indwelling and provide psychological support and nursing guidance when it is necessary to indwelling catheter due to illness | 4.84 | 0.37 | 0.08 | 84 | 4.80 | | 0.41 | 0.09 | 80 |
| A7 | Provide technical guidance and drinking water for stroke patients and caregivers who need intermittent catheterization or urination plan | 4.64 | 0.86 | 0.19 | 76 | 4.60 | | 0.65 | 0.14 | 68 |
| A8 | When leaving the hospital with tube, provide daily maintenance, spare items and record filling and other instructions | 4.76 | 0.60 | 0.13 | 84 | 4.76 | | 0.44 | 0.09 | 76 |
| B1 | The core nursing system should be strictly implemented to avoid iatrogenic injuries and economic burdens caused by improper nursing | 5.00 | 0.00 | 0.00 | 100 | 4.84 | | 0.47 | 0.10 | 88 |
| B2 | Establish a comprehensive assessment, prevention, reporting and management plan for stroke accidents and complications (such as falls, pressure sores, aspiration, increased intracranial pressure, rebleeding, deep vein thrombosis, emotional disorders, etc.) | 5.00 | 0.00 | 0.00 | 100 | 4.76 | | 0.52 | 0.11 | 80 |
| B3 | Invite patients and their families to participate in medical safety, and inform them of the prevention and treatment of stroke accidents and complications and the key points of cooperation | 4.96 | 0.20 | 0.04 | 96 | 4.76 | | 0.52 | 0.11 | 80 |
| B4 | Regardless of any operation, always adhere to the principle of minimal harm to the patient | 4.96 | 0.20 | 0.04 | 96 | 4.60 | | 0.58 | 0.13 | 64 |
| B5 | Set up eye-catching bedside cards, warning boards and safety signs | 4.88 | 0.33 | 0.07 | 88 | 4.80 | | 0.50 | 0.10 | 84 |
| B6 | The floor of the stroke ward is anti-skid, and safety facilities are fully equipped (such as convenient call bell in the activity area, stable and coherent handrails, and inductive ground lights, etc.) | 5.00 | 0.00 | 0.00 | 100 | 4.80 | | 0.41 | 0.09 | 80 |
| B7 | Flat cars, wheelchairs and other transport facilities are fully protected, and rehabilitation equipment is in good function | 4.92 | 0.28 | 0.06 | 92 | 4.80 | | 0.41 | 0.09 | 80 |
| C1 | Implementation of the first inquiry responsibility system, take the initiative to introduce the environment of stroke ward, hospitalization regulations, inform the responsible doctor and nurse's name, responsibilities, office location, and provide contact cards when necessary | 4.88 | 0.33 | 0.07 | 88 | 4.80 | | 0.50 | 0.10 | 84 |
| C2 | Establish a multi-party communication mechanism among doctors, nurses, patients and family members, and provide at least once a day and more than 5 minutes of time for communication and service feedback | 4.84 | 0.37 | 0.08 | 84 | 4.72 | | 0.46 | 0.10 | 72 |
| C3 | When there is speech disorder after stroke, the patients should agree on the expression of basic needs (such as words, pictures, gestures, etc.) in advance, and provide their caregivers with communication skills with the patients | 4.96 | 0.20 | 0.04 | 96 | 4.72 | | 0.46 | 0.10 | 72 |
| C4 | When there is cognitive impairment after stroke, patients and their families should be guided and taught the cognitive impairment management strategies (including environmental change, behavioral change, etc.) | 4.88 | 0.33 | 0.07 | 88 | 4.48 | | 0.65 | 0.15 | 56 |
| C5 | Pay attention to the fatigue status of patients with stroke, and provide fatigue related knowledge and coping skills | 4.52 | 0.92 | 0.20 | 68 | 4.44 | | 0.77 | 0.17 | 60 |
| C6 | Take the initiative to care for patients, observe and comfort patients' anxiety during treatment, and allow relatives to accompany them when necessary | 4.92 | 0.28 | 0.06 | 92 | 4.64 | | 0.64 | 0.14 | 72 |
| C7 | To assess the stress response of home caregivers and the care stress of stroke patients, and to provide channels for stress relief, care skill training, and professional escort employment | 4.92 | 0.28 | 0.06 | 92 | 4.64 | | 0.57 | 0.12 | 68 |
| C8 | Flexible visitation is allowed, providing guidance on medical treatment, dining, accommodation, shopping and activities for families in other places | 4.44 | 0.77 | 0.17 | 60 | 4.32 | | 0.75 | 0.17 | 48 |
| C9 | When the patient is not accompanied, give cordial greetings and timely company, especially on special holidays or when the patient is in mood | 4.84 | 0.37 | 0.08 | 84 | 4.76 | | 0.44 | 0.09 | 76 |
| C10 | Provide timely end-of-life education to family members, and provide space and time for emotional catharsis | 4.56 | 0.71 | 0.16 | 64 | 4.36 | | 0.86 | 0.20 | 52 |
| C11 | Organize patients' clubs and stroke caregivers' experience sharing sessions to provide interaction between patients and caregivers | 4.76 | 0.44 | 0.09 | 76 | 4.56 | | 0.65 | 0.14 | 64 |
| D1 | Abide by the professional image and code of conduct, in words and deeds to reflect the patient's personal dignity, informed consent, autonomy, privacy, cultural customs and other respect | 4.92 | 0.28 | 0.06 | 92 | 4.84 | | 0.37 | 0.08 | 84 |
| D2 | Provide a relatively independent place for doctor-patient communication and informed information | 4.80 | 0.41 | 0.09 | 80 | 4.64 | | 0.57 | 0.12 | 68 |
| D3 | Patients are invited to participate in the whole process of medical decision-making, and jointly participate in the formulation of rehabilitation goals, rehabilitation content and discharge plan | 4.72 | 0.54 | 0.11 | 76 | 4.56 | | 0.65 | 0.14 | 64 |
| D4 | Patients with limited or slow movement should be provided with sufficient patience | 4.84 | 0.37 | 0.08 | 84 | 4.68 | | 0.56 | 0.12 | 72 |
| D5 | Be polite to the patient at all times and do not call the bed number directly | 4.84 | 0.37 | 0.08 | 84 | 4.88 | | 0.44 | 0.09 | 92 |
| D6 | Do not use disrespectful words or behaviors due to the patient's disability and pathological stress response (such as sexual response, post-stroke fatigue, post-stroke emotional disorder, urinary and fecal incontinence, etc.) | 4.96 | 0.20 | 0.04 | 96 | 4.76 | | 0.44 | 0.09 | 76 |
| D7 | Maximally meet the reasonable needs of patients, when they can not take care of themselves, help to maintain their personal image, give timely help and guide the development of self-care ability training | 4.88 | 0.33 | 0.07 | 88 | 4.60 | | 0.58 | 0.13 | 64 |
| D8 | Reasonable charge, provide daily charge list | 4.28 | 1.17 | 0.27 | 60 | 4.64 | | 0.70 | 0.15 | 76 |
| E1 | The multidisciplinary team worked together to develop a personalized discharge plan according to the patient's condition, neurological status, risk factor management status and other conditions | 4.76 | 0.52 | 0.11 | 80 | 4.52 | | 0.65 | 0.14 | 60 |
| E2 | To guide the development of early rehabilitation, and inform the patients and their families of the key points of cooperation in this process | 4.84 | 0.47 | 0.10 | 88 | 4.56 | | 0.65 | 0.14 | 64 |
| E3 | To help patients correctly recognize the rehabilitation goals of stroke at all stages, to build rehabilitation confidence, to encourage timely progress when there is progress, reasonable counseling when the effect is not ideal, to improve rehabilitation compliance | 4.92 | 0.28 | 0.06 | 92 | 4.68 | | 0.48 | 0.10 | 68 |
| E4 | To provide a good rehabilitation training environment, industrial and recreational rooms and convenient service facilities (e.g. rehabilitation equipment, transfer equipment, etc.) | 4.84 | 0.37 | 0.08 | 84 | 4.52 | | 0.59 | 0.13 | 56 |
| E5 | Provide a variety of rehabilitation programs (such as Chinese medicine), rehabilitation skills training guidance (such as: education leaflets, rehabilitation videos, etc.) | 4.84 | 0.47 | 0.10 | 88 | 4.52 | | 0.65 | 0.14 | 60 |
| E6 | Upon admission, the patient was screened for swallowing, speech, hearing, psychological and sleep disorders and followed up continuously. When necessary, the patient was referred to a specialist for further evaluation to ensure that the patient could get professional and comprehensive rehabilitation treatment | 4.92 | 0.28 | 0.06 | 92 | 4.72 | | 0.54 | 0.11 | 76 |
| E7 | Specialized personnel shall conduct whole-process management and follow-up of patients' health conditions, distribute individualized health education prescriptions, continuously conduct health education, guide the management of stroke risk factors and improve medication compliance | 4.80 | 0.41 | 0.09 | 80 | 4.56 | | 0.65 | 0.14 | 64 |
| E8 | Provide professional medical information and technical guidance throughout the process (such as stroke prevention, stroke standard diagnosis and treatment, stroke rehabilitation, medical insurance, occupational counseling, psychological services, etc.) | 4.88 | 0.33 | 0.07 | 88 | 4.56 | | 0.58 | 0.13 | 60 |
| E9 | Before discharge, we will provide on-site visits according to the needs, and give home modification and home care guidance | 4.64 | 0.57 | 0.12 | 68 | 4.20 | | 0.91 | 0.22 | 40 |
| E10 | Provide communication channels and information to community rehabilitation, social welfare, social work groups, social resources, undertakers and other institutions before discharge or when necessary | 4.60 | 0.71 | 0.15 | 68 | 4.28 | | 0.98 | 0.23 | 56 |
| E11 | Provide continued care and follow-up after discharge, regular follow-up and continuous supervision (including medication and rehabilitation compliance, risk factor management, return visit, etc.) | 4.84 | 0.37 | 0.08 | 84 | 4.56 | | 0.58 | 0.13 | 60 |
| Second round | | | | | | | | | | |
| A1 | Screening for dysphagia was completed within 24 hours after admission, and nutritional assessment was performed within 48 hours for those with positive results | 4.96 | 0.20 | 0.04 | 96 | 4.68 | | 0.56 | 0.12 | 72 |
| A2 | Develop targeted dietary guidance and eating care based on patient's eating habits, swallowing and nutrition assessment results (including: tableware and food selection, food form adjustment, bite-size principle, compensatory approach, oral management, etc.) | 5.00 | 0.00 | 0.00 | 100 | 4.88 | | 0.33 | 0.07 | 88 |
| A3 | Instruction of patients in bed excretion skills and prevention of constipation and diarrhea, when constipation and diarrhea should be timely intervention | 4.80 | 0.50 | 0.10 | 84 | 4.88 | | 0.33 | 0.07 | 88 |
| A4 | Inform the use of diapers, urine collection bags and other auxiliary equipment, and improve the nursing equipment according to the actual needs when necessary | 4.68 | 0.63 | 0.13 | 76 | 4.76 | | 0.44 | 0.09 | 76 |
| A5 | Sleep time to limit visits, treatment operations are centralized, light is soft, avoid direct eye | 4.68 | 0.63 | 0.13 | 76 | 4.40 | | 0.76 | 0.17 | 56 |
| A6 | Maintain the patient's physical comfort, assess his pain and agitation index, eliminate or reduce pain and adverse irritation in time | 4.84 | 0.47 | 0.10 | 88 | 4.64 | | 0.57 | 0.12 | 68 |
| A7 | Teach the patient to take the medicine correctly (e.g. time, dosage, usage, adverse effects, etc.) and let him know | 4.88 | 0.33 | 0.07 | 88 | 4.72 | | 0.54 | 0.11 | 76 |
| A8 | Minimize unnecessary catheter indwelling and provide psychological support and nursing guidance when it is necessary to indwelling catheter due to illness | 4.80 | 0.41 | 0.09 | 80 | 4.56 | | 0.65 | 0.14 | 64 |
| A9 | When leaving the hospital with the tube, provide the necessary guidance for daily maintenance, reserve items acquisition, record filling and so on | 4.80 | 0.41 | 0.09 | 80 | 4.68 | | 0.48 | 0.10 | 68 |
| B1 | According to the rapid identification method of stroke, the symptoms of stroke can be accurately identified, and the green channel of stroke can be activated with one button, and the whole treatment process can be smoothly connected | 4.96 | 0.20 | 0.04 | 96 | 4.84 | | 0.37 | 0.08 | 84 |
| B2 | The green channel system of stroke in our hospital was improved according to national standards, and continuous quality improvement was carried out | 4.92 | 0.28 | 0.06 | 92 | 4.84 | | 0.37 | 0.08 | 84 |
| B3 | We will strictly implement the core medical system and nursing operation procedures to avoid iatrogenic injuries and economic burdens caused by improper nursing | 4.96 | 0.20 | 0.04 | 96 | 4.96 | | 0.20 | 0.04 | 96 |
| B4 | Improve the evaluation, prevention, reporting and management of stroke related complications and adverse events and implement them through training (such as: muscle spasm, aspiration, rebleeding, postoperative hematoma, fall and fall, stress injury, unplanned extubation, self-injury and suicide, etc.) | 5.00 | 0.00 | 0.00 | 100 | 4.88 | | 0.33 | 0.07 | 88 |
| B5 | Under the premise of the same curative effect, choose the treatment and nursing plan with the least harm, the least pain and the least cost | 4.84 | 0.37 | 0.08 | 84 | 4.72 | | 0.46 | 0.10 | 72 |
| B6 | Invite patients and caregivers to participate in medical safety, and inform them of the prevention and treatment points of stroke accidents and complications | 4.96 | 0.20 | 0.04 | 96 | 4.72 | | 0.46 | 0.10 | 72 |
| B7 | Pay attention to the feedback of patients and caregivers, patrol the ward in time, and deal with their problems and potential physical, psychological, environmental and other safety hazards in time | 4.80 | 0.41 | 0.09 | 80 | 4.68 | | 0.48 | 0.10 | 68 |
| B8 | Set up eye-catching bedside cards, warning signs and safety signs, and educate patients and caregivers about their significance and effects | 5.00 | 0.00 | 0.00 | 100 | 4.96 | | 0.20 | 0.04 | 96 |
| B9 | The floor of the stroke ward is anti-skid, and safety facilities are fully equipped (such as convenient call bell in the activity area, stable and coherent handrails, and inductive ground lights, etc.) | 5.00 | 0.00 | 0.00 | 100 | 4.84 | | 0.37 | 0.08 | 84 |
| B10 | Flat car, wheelchair, rehabilitation equipment and other equipment is 100% perfect, safety protection measures are complete | 4.92 | 0.28 | 0.06 | 92 | 4.88 | | 0.33 | 0.07 | 88 |
| C1 | Reduce the environmental strangeness of new patients, take the initiative to complete admission education, and inform them of the names, responsibilities and office locations of doctors and nurses in charge of beds, and provide medical contact cards if necessary | 4.92 | 0.28 | 0.06 | 92 | 4.84 | | 0.37 | 0.08 | 84 |
| C2 | Establish a communication mechanism among doctors, nurses and patients, and provide at least once a day and more than 5 minutes of time for communication and service feedback | 4.92 | 0.28 | 0.06 | 92 | 4.80 | | 0.41 | 0.09 | 80 |
| C3 | Assist patients with speech disorders after stroke to express the needs of daily life and teach compensatory communication skills (such as gestures, writing, mouth movements, tablets, mobile phones and other media), and use non-verbal communication when necessary to make patients feel care (such as tapping patients on the shoulder, shaking hands and other therapeutic caressing). | 4.84 | 0.37 | 0.08 | 84 | 4.80 | | 0.50 | 0.10 | 84 |
| C4 | To understand the most concerned problems of patients and help them solve them, listen to their emotional expression patiently, comfort the bad emotions in the process of diagnosis and treatment, use anxiety, depression and other scales to screen post-stroke emotional disorders, and refer them to the psychiatric department when necessary | 4.96 | 0.20 | 0.04 | 96 | 4.84 | | 0.37 | 0.08 | 84 |
| C5 | Take the initiative to care for patients. When patients are not accompanied, give them cordial greetings and timely company, especially during special holidays or mood changes | 5.00 | 0.00 | 0.00 | 100 | 4.96 | | 0.20 | 0.04 | 96 |
| C6 | Assist in the establishment of a family support system, actively communicate with family members about the patient's recovery progress, and ensure that they understand and accept the patient's mental and physical state after stroke | 4.96 | 0.20 | 0.04 | 96 | 4.84 | | 0.37 | 0.08 | 84 |
| C7 | To assess the stress response of family caregivers and the care stress of stroke patients, and to provide channels for stress relief, care skill training, and professional escort employment | 4.92 | 0.28 | 0.06 | 92 | 4.68 | | 0.48 | 0.10 | 68 |
| C8 | Flexible visitation is allowed appropriately, and guidance on dining, accommodation, shopping and activities is provided for families from other places | 4.68 | 0.56 | 0.12 | 72 | 4.52 | | 0.51 | 0.11 | 52 |
| C9 | Family members of dying patients should be provided with hospice care education at the right time to provide space and time for emotional catharsis for those in need | 4.84 | 0.37 | 0.08 | 84 | 4.60 | | 0.58 | 0.13 | 64 |
| C10 | Organize patients' clubs and experience sharing sessions for stroke caregivers to provide a platform for communication between patients and caregivers | 4.84 | 0.37 | 0.08 | 84 | 4.80 | | 0.41 | 0.09 | 80 |
| D1 | Abide by the professional image and code of conduct, and reflect the respect for patients' informed consent, autonomy, privacy and cultural customs in nursing services | 5.00 | 0.00 | 0.00 | 100 | 4.96 | | 0.20 | 0.04 | 96 |
| D2 | Use polite and appropriate terms of address, and never call the patient by his or her bed number | 5.00 | 0.00 | 0.00 | 100 | 5.00 | | 0.00 | 0.00 | 100 |
| D3 | Establish a relatively independent doctor-patient communication and informed place | 4.88 | 0.33 | 0.07 | 88 | 4.80 | | 0.41 | 0.09 | 80 |
| D4 | Make clear the treatment and care plan, and encourage the patient and family to participate in medical decisions and fully respect their wishes | 4.96 | 0.20 | 0.04 | 96 | 4.80 | | 0.41 | 0.09 | 80 |
| D5 | Understand the patient's situation and disease status, be patient in case of slow or inconvenient movement, and do not discriminate because of physical disability or stress reaction (such as sexual reaction, post-stroke fatigue, post-stroke emotional disorder, urinary and bowel incontinence, etc.) | 4.96 | 0.20 | 0.04 | 96 | 4.92 | | 0.28 | 0.06 | 92 |
| D6 | Maximally meet the reasonable needs of patients, when they can not take care of themselves, timely help to assist in maintaining personal image, and guide the development of self-care ability training | 4.92 | 0.28 | 0.06 | 92 | 4.88 | | 0.33 | 0.07 | 88 |
| E1 | The multidisciplinary team worked together to develop a personalized discharge plan based on the patient's condition, neurological status, risk factor management and other conditions | 4.92 | 0.28 | 0.06 | 92 | 4.84 | | 0.37 | 0.08 | 84 |
| E2 | To help patients correctly understand the phased rehabilitation goals of stroke rehabilitation, to build rehabilitation confidence, to encourage timely progress when there is progress, reasonable counseling when the effect is not ideal, to improve rehabilitation compliance | 4.96 | 0.20 | 0.04 | 96 | 4.88 | | 0.33 | 0.07 | 88 |
| E3 | Guide patients and caregivers to carry out early rehabilitation correctly, and inform them of the operation and cooperation points in the training process | 4.96 | 0.20 | 0.04 | 96 | 4.92 | | 0.28 | 0.06 | 92 |
| E4 | Provide a suitable rehabilitation training environment, a variety of rehabilitation programs (such as appropriate Chinese medicine techniques, rehabilitation therapy equipment, etc.) and convenient service facilities (such as rehabilitation equipment, transport equipment, etc.) | 4.92 | 0.28 | 0.06 | 92 | 4.60 | | 0.58 | 0.13 | 64 |
| E5 | Complete management and follow-up of patients' health conditions, individualized health education prescriptions, continuous health education, guidance and management of stroke risk factors and improvement of medication compliance | 4.92 | 0.28 | 0.06 | 92 | 4.80 | | 0.41 | 0.09 | 80 |
| E6 | Pay attention to the fatigue state of patients after stroke, evaluate the severity of fatigue and provide coping strategies | 4.88 | 0.33 | 0.07 | 88 | 4.76 | | 0.44 | 0.09 | 76 |
| E7 | When there is cognitive impairment after stroke, cognitive function training should be carried out, and caregivers should be provided with management guidance related to cognitive impairment (including environmental change, behavioral change, etc.). | 4.92 | 0.28 | 0.06 | 92 | 4.76 | | 0.44 | 0.09 | 76 |
| E8 | Provide professional medical information and technical guidance (e.g. stroke prevention, stroke rehabilitation, occupational counseling, psychological support, medication and health insurance, etc.) | 4.92 | 0.28 | 0.06 | 92 | 4.84 | | 0.37 | 0.08 | 84 |
| E9 | Before discharge, home care and home modification guidance were provided, and home visits were provided when possible | 4.76 | 0.44 | 0.09 | 76 | 4.56 | | 0.71 | 0.16 | 68 |
| E10 | Provide communication channels and information for community rehabilitation, social welfare, social work groups, social resources, funeral services, etc | 4.76 | 0.44 | 0.09 | 76 | 4.56 | | 0.65 | 0.14 | 64 |
| E11 | Provide continued care and follow-up after discharge, regular follow-up and continuous supervision (including medication and rehabilitation compliance, risk factor management, return visit, etc.) | 4.96 | 0.20 | 0.04 | 96 | 4.80 | | 0.41 | 0.09 | 80 |
